# 3D-MCN: A 3D Multi-scale Capsule Network for Lung Nodule Malignancy Prediction

**DOI:** 10.1038/s41598-020-64824-5

**Published:** 2020-05-14

**Authors:** Parnian Afshar, Anastasia Oikonomou, Farnoosh Naderkhani, Pascal N. Tyrrell, Konstantinos N. Plataniotis, Keyvan Farahani, Arash Mohammadi

**Affiliations:** 1 Concordia Institute for Information Systems Engineering, Montreal, QC Canada; 2 Department of Medical Imaging, Sunnybrook Health Sciences Centre, University of Toronto, Toronto, ON Canada; 3 Department of Medical Imaging, Department of Statistical Sciences, University of Toronto, Toronto, ON Canada; 4 Department of Electrical and Computer Engineering, University of Toronto, Toronto, ON Canada; 5 Center for Biomedical Informatics and Information Technology, National Cancer Institute (NCI), Rockville, MD USA

**Keywords:** Cancer imaging, Non-small-cell lung cancer

## Abstract

Despite the advances in automatic lung cancer malignancy prediction, achieving high accuracy remains challenging. Existing solutions are mostly based on Convolutional Neural Networks (CNNs), which require a large amount of training data. Most of the developed CNN models are based only on the main nodule region, without considering the surrounding tissues. Obtaining high sensitivity is challenging with lung nodule malignancy prediction. Moreover, the interpretability of the proposed techniques should be a consideration when the end goal is to utilize the model in a clinical setting. Capsule networks (CapsNets) are new and revolutionary machine learning architectures proposed to overcome shortcomings of CNNs. Capitalizing on the success of CapsNet in biomedical domains, we propose a novel model for lung tumor malignancy prediction. The proposed framework, referred to as the 3D Multi-scale Capsule Network (3D-MCN), is uniquely designed to benefit from: (i) 3D inputs, providing information about the nodule in 3D; (ii) Multi-scale input, capturing the nodule’s local features, as well as the characteristics of the surrounding tissues, and; (iii) CapsNet-based design, being capable of dealing with a small number of training samples. The proposed 3D—MCN architecture predicted lung nodule malignancy with a high accuracy of 93.12%, sensitivity of 94.94%, area under the curve (AUC) of 0.9641, and specificity of 90% when tested on the LIDC-IDRI dataset. When classifying patients as having a malignant condition (i.e., at least one malignant nodule is detected) or not, the proposed model achieved an accuracy of 83%, and a sensitivity and specificity of 84% and 81% respectively.

## Introduction

According to recent statistics on cancer types published by the International Agency for Research on Cancer of the World Health Organization (WHO)^1^, lung cancer is ranked first worldwide for mortality and is among the top three cancer types for incidence. Lung cancer together with breast cancer lead worldwide for the number of new cases with approximately 2.1 million diagnoses estimated in 2018. Lung cancer is also responsible for the largest number of deaths (1.8 million deaths, 18.4% of the total), with a low 5-year survival rate (18%)^2^. This high mortality rate is mainly due to the fact that lung cancer is diagnosed in more than half of the cases at advanced stages^2^. In recent years, significant technological advancements in medical imaging, especially Computed Tomography (CT), have improved the detection rate of lung tumors^3^. Analyzing and interpreting these images, however, is time consuming^4^, and subject to inter-observer variability. Furthermore, intrinsic tumor heterogeneity can significantly contribute to the cancer diagnosis and may not be always visible to the human eye^5^.

The aforementioned shortcomings of human-centered cancer diagnosis have encouraged the development of a relatively new field in medical imaging, referred to as “radiomics”^6–8^, which has shown promising results in cancer diagnosis^9^. Radiomics is the machine extraction of hundreds of quantitative and semi-quantitative features from radiographic images^10^ based on the hypothesis that these features can provide more information to the unaided eye. The goal of radiomics analysis, in cancer, is to improve detection, diagnosis, or prediction of the treatment outcome in each case. Radiomics analysis has been recently categorized in two main groups: namely hand-crafted and deep learning-based radiomics. In the former category^11–14^, a set of pre-defined engineered features are extracted from medical images. These features are, consequently, passed to an analysis tool to calculate the desired output such as the probability of cancer, its stage, and/or the estimated survival time. Although hand-crafted features have shown considerable correlation with clinical outcomes, they have several weaknesses^15^ limiting their applicability and reliability. First, while hand-crafted features can be stable and robust in some tasks, this group of features is, usually, extracted from the annotated region of interest, which can only be provided by the experts. Therefore, not only does their calculation require extensive time and effort but also their stability and reproducibility highly depend on the pre-processing steps and segmentation^16^. Second, hand-crafted radiomics are pre-defined and need prior knowledge on the types of features to be extracted, which is often unavailable. Furthermore, there is no guarantee that the extracted features are useful for the task at hand (cancer prediction, diagnosis, and/or classification), as defining the features is independent from the analysis process.

The shortcomings of the hand-crafted radiomics have led to the emergence of the deep learning-based radiomics^17–20^ where features are not pre-defined. In fact, deep learning-based radiomic features are learned in an end-to-end fashion while taking specifics of the desired output into account. In other words, the model learns to extract features that can best distinguish the classes. Convolutional Neural Networks (CNNs) are one of the most popular deep networks in the field of radiomics^21^. These networks benefit from several useful properties^22^; for example, sharing trainable weights over the input that significantly reduces the training time and enables the model to extract local features from the whole input image. The CNNs, however, require large datasets, and cannot generalize well in the absence of a large number of images, which is usually the of case a clinical cohort of patients^23^ and included in the problem of lung cancer classification. Another limitation is the “black box” model, which does not allow the researcher to identify which features are actively playing a role in the classification or if there are any differences between the impact of the features in subgroups of the population. Determining positive or negative samples may be sufficient but often the radiologist will contribute to treatment decisions that depend on more than identifying cancer. For instance, depending on texture, tumor size, and growth rate, various treatment options will be considered for the patient.

Capsule networks ^24^ (also referred to as CapsNets) are relatively new and revolutionary deep learning architectures that are capable of identifying spatial relations between different objects in an image. They eliminate the need for a large number of samples by including all the possible rotations and transformations of the underlying objects. Therefore, CapsNets are potentially applicable in medical domains^25^, where very large datasets are unavailable. In our recent works^25,26^, which can be considered the first attempts at applying CapsNets in a biomedical domain, Capsule networks were designed for the purpose of brain tumor type classification via magnetic resonance imaging (MRI) data. Capitalizing on the success of CapsNets for brain tumor-type classification and the importance of lung cancer malignancy prediction, we propose in this paper a novel and advanced CapsNet architecture designed specifically for lung malignancy prediction based on computed tomography (CT). The proposed 3D multi-scale CapsNet (3D—MCN) model takes 3D patches of the nodules at three different scales as inputs and predicts the nodule’s malignancy. The rationale is that the morphological characteristics of the nodule are not the only ones predicting its malignancy, and incorporation of information obtained from the surrounding tissues and vessels play a critical role in determining the type of the nodule. In brief, the 3D—MCN benefits from: (i) *CapsNets-based design*, utilizing unique characteristics of this revolutionary deep learning model in handling small datasets and object transformations; (ii) *3D inputs*, which give the model access to 3D features of the nodule; (iii) *multi-scale inputs*, helping the CapsNet to assess the local and global features, and; (iv) not requiring the nodule detailed annotation and pre-defined features.

The aim of this work is to investigate whether the proposed 3D-MCN can solve several fundamental challenges of lung nodule malignancy prediction: the first is that most of the traditional nodule prediction models required an exact annotation, leading to inter-observer variability, along with features that are not consistent and reproducible; the second is the insufficiency of the features extracted from the nodule region alone. The surrounding tissues, containing valuable information for the nodule prediction, are carefully taken into account in the 3D-MCN; the third is to assess whether the capsule network framework, embedded in the 3D-MCN, can operate efficiently without requiring a large dataset while yielding acceptable diagnostic accuracy. Finally, the last challenge to address is the model’s interpretability, for which the 3D-MCN features and their correlations with features used in clinical practice are investigated as building trust between the model and its clinical users is of paramount importance.

## Results

### Proposed 3D-MCN Model for lung nodule classification

3D-MCN consists of three independent CapsNets, each of which takes nodule patches at a different spatial scale as input. Therefore, we refer to it as a multi-scale learning architecture. Here, scale refers to the visible area of the tissue surrounding the nodule. Each input is a 3D nodule crop centered at the nodule annotation. The output vectors of the three CapsNets are concatenated, and the result goes through a fusion module consisting of a set of fully connected layers. The final output is the probability of the nodule being benign or malignant.

**Figure 1:**
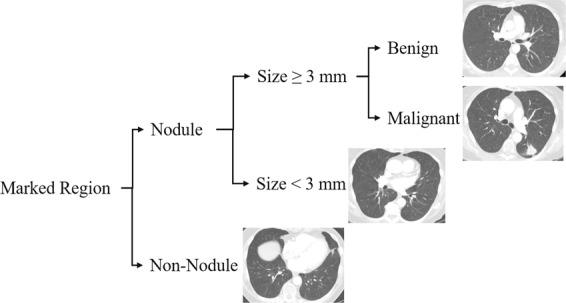
Examples of available marked regions in the LIDC-IDRI dataset^27^. Each region was classified as nodule or non-nodule. Nodules were categorized based on their size. Nodules larger than 3 *mm* were further sub-categorized based on their malignancy ratings.

We performed several experiments on two independent training and test sets from the Lung Image Database Consortium (LIDC) and Image Database Resource Initiative (IDRI) dataset^27–29^. This dataset consisted of the CT scans of 1018 subjects. The images were annotated and labeled by one to four radiologists. Each marked region was either nodule or non-nodule. Nodule cases were further divided into two categories. The first category contained the nodules that were less than 3 mm in size. The second category included those that were equal to or larger than 3 mm. For the latter category, radiologists identified the degree of malignancy on a scale of 1 to 5, where the larger the number, the higher the possibility of malignancy. In this work, we categorized nodules 3 mm or larger as either benign (low degree of malignancy) or malignant (high degree of malignancy). Figure 1 shows illustrative examples of available marked regions in the dataset used for evaluation purposes of the proposed 3D—MCN framework.

### Performance of the proposed 3D multi-scale CapsNet (3D—MCN)

Among the five available malignancy labels in the dataset, we followed recent studies^5,30–33^ and decided to discard the nodules for which the average malignancy ratings given by the radiologists (rounded to the nearest integer) was 3 (indeterminate malignancy). The remaining four labels were divided into two groups, where labels 1 and 2 were re-labeled as 0 (unlikely to be malignant), and labels 4 and 5 were re-labeled as 1 (likely to be malignant). Therefore, a binary classification problem was constructed for a total of 2283 nodules. Nodules were, consequently, grouped into two independent sets for testing (30%) and training (70%). We confirmed that there were no shared patients between the two underlying sets. We trained the single-scale models and the proposed multi-scale architecture based on the training set, and evaluated its performance over the test set. In addition, we trained a fully-connected neural network (having the same architecture as the one in the multi-scale model), as the base-line model, on four hand-crafted features; namely volume, diameter, center-of-mass *x* coordinate, and center-of-mass *y* coordinate. These four features accompany the IDC-IDRI dataset to ensure all research groups use the same size-selected nodules. To have a more insightful comparison, we also designed and implemented a 3D-CNN architecture that had inputs at the three scales (similar in nature to the 3D-MCN but without the Capsule design). We tried to keep the complexity of the 3D-CNN as similar as possible to the proposed 3D-MCN. We utilized three independent CNNs, each of which was trained on a specific scale. The final layer of all the three CNNs was a softmax layer to predict the lung tumor malignancy. However, to be able to combine the CNNs before making the final decision, for all the three networks, we took the layer before the softmax, consisting of 32 nodes (similar to the dimension of the final capsule in the proposed 3D-MCN), and concatenated them to go through the fully connected network. The details of the implemented 3D-CNN architecture was as follows:

- The input to each CNN was a 3D lung nodule patch at a specific scale, similar to the setting utilized in the 3D-MCN.
- The first layer was a convolutional one, with filters of size 9 and stride of 1, outputting 256 feature maps.
- The second layer was a convolutional one, with filters of size 9 and stride of 2, outputting 256 feature maps.
- The third layer was a fully-connected one, with 32 nodes.
- The final layer consisted of two nodes to decide on the malignancy of the lung nodule.
- CNNs at different scales were trained independently.
- For all three CNNs, the layers before the final one were concatenated.
- The resulting vector went through a set of fully-connected layers, as utilized in the 3D-MCN.
- The final fully-connected layer made the final decision.

The designed 3D-CNN was trained and tested on exactly the same training and test sets as used for the evaluation of the 3D-MCN.

**Figure 2:**
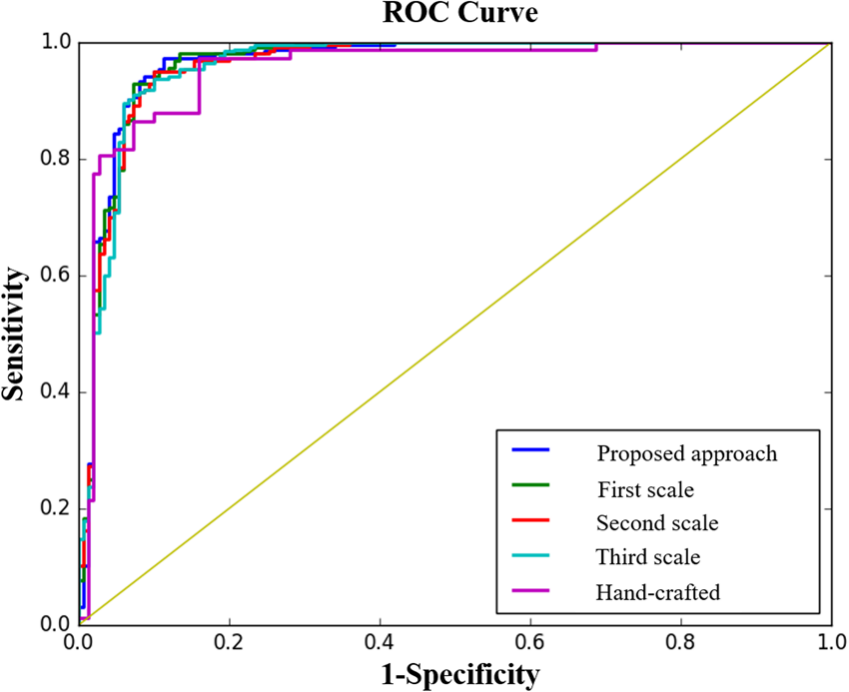
The ROC curve for the single-scale, multi-scale, and the base-line model (i.e., fully connected layers trained on hand-crafted features only), showing that the proposed 3D−MCN approach was capable of achieving the highest AUC.

Four measurements based on the performance of the test set were calculated: area under the curve (AUC), accuracy, specificity, and sensitivity. The results are provided in Table 1, which shows that the 3D—MCN framework outperforms not only the single-scale models, but also the 3D-CNN, which takes the exact multi-scale patches as inputs. Moreover, even the single-scale Capsule models outperformed the 3D-CNN, which further illustrates on the superiority of the Capsule design. Specificity was higher for the second scale model. However, we believe that sensitivity is of greater importance, as the consequences of misclassification are worse for malignant cases. Furthermore, typically in clinical practice, suspected malignant cases will go over complementary examinations^34^, which can identify whether the underlying case was a false positive. Figure 2 illustrates the Receiver Operating Characteristic (ROC) curve for the single-scale models, as well as the multi-scale one, and the base-line.

**Table 1:** Performance of the proposed 3D Multi-scale CapsNet along with performance of the three underlying single scale-models and the described 3D-CNN on the independent test set. The 3D—MCN approach outperformed others in terms of the AUC, accuracy, and sensitivity.

Model	Area Under the Curve (AUC)	Accuracy	Specificity	Sensitivity
Proposed 3D—MCN	**0.964**	**93.12%**	90%	**94.94%**
First Scale of the 3D—MCN	0.9633	91.65%	90%	92.21%
Second Scale of the 3D—MCN	0.96	91.65%	**91.33%**	91.82%
Third Scale of the 3D—MCN	0.96	91.40%	89.33%	92.60%
3D-CNN	0.9562	89.43%	90%	89.10%
Base-line Model (Hand-Crafted)	0.9524	87.47%	86.66%	87.93%

In clinical applications, where false positives and false negatives are not treated equally, a threshold can be selected based on the ROC curve to set a desired sensitivity and specificity. Another strategy to tune these measures is to assign different weights in the objective function. In this work, the proposed 3D multi-scale CapsNet was trained with the objective of minimizing the binary cross entropy loss^35,36^. As such, we modified the loss as follows to put different weights on the loss function terms (specificity and sensitivity)

1 $${\rm{l}}{\rm{o}}{\rm{s}}{\rm{s}}=-\,\,\,\,\,\,\mathop{\underbrace{(\alpha \,y\,log(p)}}\limits_{\text{Controlling false positive}}+\,\mathop{\underbrace{\beta (1-y)\,log(1-p)}}\limits_{\text{Controlling false negative}}),$$

where *y* is the target, and *p* is the predicted probability of Class 1. Terms *α* and *β* denote the weights given to the false positives and false negatives, respectively. We trained the multi-scale model with three settings: equal weights, more weight assigned to the false positive, and more weight given to the false negative. Table 2 shows the obtained results.

**Table 2:** Effects of changing the weights associated with the terms in the modified loss function controlling the false positives and false negatives. Assigning more weight to the false positive loss increased the sensitivity, while putting higher weight to the false negative loss increased the specificity.

Weight Setting	Area Under the Curve (AUC)	Accuracy	Specificity	Sensitivity
Equal Weights	_0.964_	_93.12%_	_90%_	_94.94%_
_*α*=2_ and *β*=1	_0.9641_	_93.12%_	_87.33%_	_96.49%_
_*α*=1_ and *β*=2	_0.9638_	_92.87%_	_92%_	_93.38%_

We inspected the cases in the test set for which our proposed approach failed to predict the correct label. We observed that 28% of such failure cases were nodules that were marked by only one radiologist. There was no agreement on these cases being nodules between different radiologists. Although all other failure cases were nodules identified by at least two radiologists, there was a common pattern between most of them, i.e., the malignancy labels were not consistent, and moreover, there was at least one label 3 among the provided labels. In other words, although the average malignancy was not 3 to be discarded, there was a high probability that the malignancy status of the nodule cannot be determined (malignancy is indeterminate).

**Figure 3:**
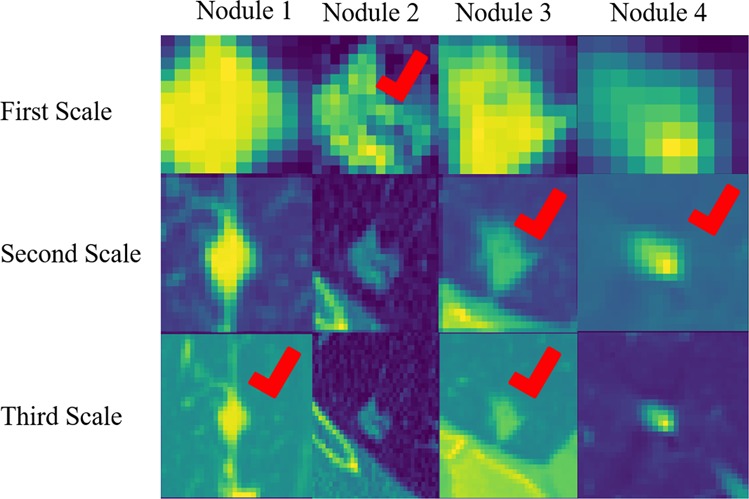
Cases, where not all the single-scale models provided correct predictions. The check sign indicates the successful scale. This figure illustrates the necessity of including all the three scales in the final model.

As stated previously, the motivation behind our multi-scale approach was that the morphological characteristics of the nodule were not the only indicators of its malignancy. In fact, the surrounding tissues and vessels played an important role in determining the benign or malignant status of the nodule^37^. To further illustrate the importance of having multi-scale inputs, we extracted the cases where the output was different for different scales. Figure 3 presents four nodules from three different scales. The figure also indicates the scale which has been successful in classifying the nodule. Having a correct prediction was not possible without including all the scales.

**Figure 4:**
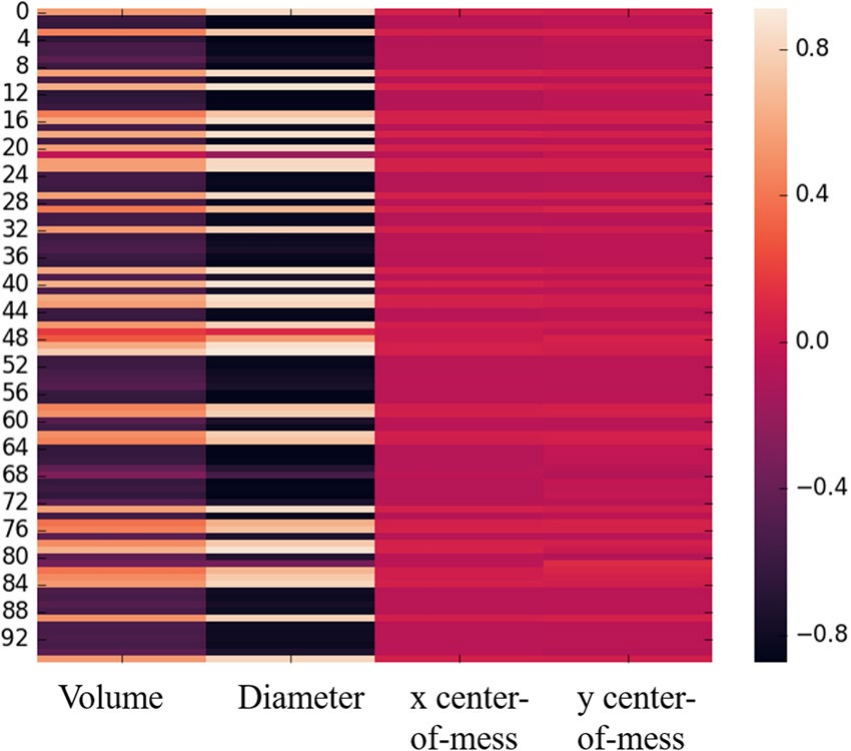
Correlation between the CapsNet features and the hand-crafted features. Most of the features were positively or negatively correlated with volume and diameter. However, the correlation with *x* and *y* centers was low, as the model was fed with cropped nodule slices in different scales, and the location with respect to the whole image was not accessible to the model.

In another experiment, we calculated the correlation between the CapsNet-extracted features from all three scales with the four hand-crafted features of volume, diameter, *x* center-of-mass, and *y* center-of-mass, as shown in Fig. 4. Volume and diameter are important factors of the nodule malignancy. Therefore, most of the learned features were highly (positively or negatively) correlated with these two features. The centers of mass are, however, calculated from the whole images, and as the model was being fed with only the cropped nodule slices via different scales, the learned features could not represent these two characteristics.

**Figure 5:**
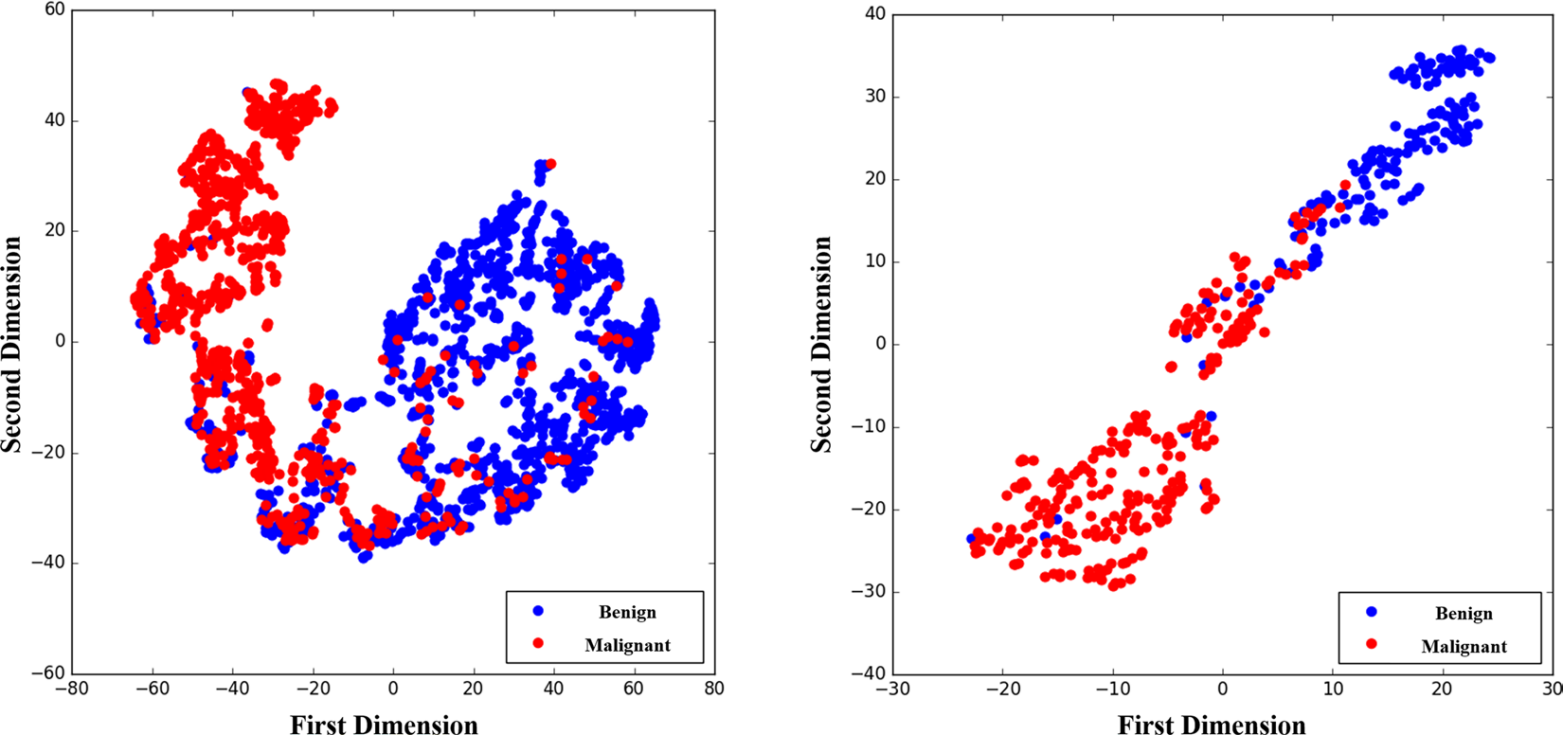
T-SNE plot of the CapsNet learned features in 2D,showing that the features were capable of distinguishing between the two classes.

When extracting deep learning-based radiomics features, it is crucially important to consider their capability in distinguishing the classes. We projected the high dimensional feature space of the CapsNet into a lower dimensional space, using a *t*-Distributed Stochastic Neighbor Embedding (*t*-SNE)^38^. The resulting feature space for both the training and test sets are shown in Fig. 5, according to which, features are distinctive even in the simplified 2D space.

### Patient-level classification

In clinical practice, radiologists label a patient as having a malignancy when even one malignant tumor is detected. Therefore, we tested the performance of the 3D—MCN model, in classifying patients. We labeled patients in the test set as having malignancy or not, where not having malignancy refers to a patient whose detected nodules are all benign. The accuracy of the 3D—MCN model in classifying patients was 83%, and sensitivity and specificity were 84% and 81% respectively.

## Discussion

In this paper, we presented our work on lung nodule malignancy prediction based on a 3D multi-scale capsule network. Our results showed that the proposed 3D—MCN approach can reach higher accuracy compared to previous studies. The model has several properties that improved the performance of the nodule malignancy prediction task including: (i) incorporation of 3D inputs to ensure that all the available information about the nodule was being used; (ii) three independent networks were trained on three different spatial scales to ensure that the network had access to not only the local features of the nodule and its shape, but also the extended features extracted from the surrounding tissues (having multi-scale input becomes more important when the radiologist has reviewed the tumor with a knowledge of the other nodules and surrounding tissues); (iii) The main architecture was the CapsNet, which, in contrast to CNNs, is able to handle smaller datasets and it is less prone to over-fitting; and (iv) the 3D—MCN framework did not rely on the hand-crafted features and detailed annotations, resulting in time-efficiency, and was not subject to inter-observer variability.

We explored different scenarios of using the LIDC-IDRI dataset for lung nodule malignancy prediction before arriving at the final model. First,we considered each 2D slice of the nodule as a separate sample, where the assigned label was the malignancy label of the corresponding nodule. This technique, although having the advantage of increasing the size of the dataset, was not successful, as whole nodules were not well represented in single 2D slices. Second, we considered only the central slice (slice taken from the middle of the nodule) as the input to the network. This strategy also failed to generate satisfactory predictions since one single slice cannot provide enough information about the nodule. However, it is faster because 2D inputs require far less trainable parameters compared to the 3D ones. Third, We tried single-scale instead of the multi-scale models, resulting in relatively poorer performance. Finally, for each identified nodule by any of the radiologists, we used the label provided by that radiologist instead of averaging over all the ratings of the corresponding nodules. This approach was also not as successful as the proposed 3D—MCN, since the network cannot handle the existing inconsistencies.

**Figure 6:**
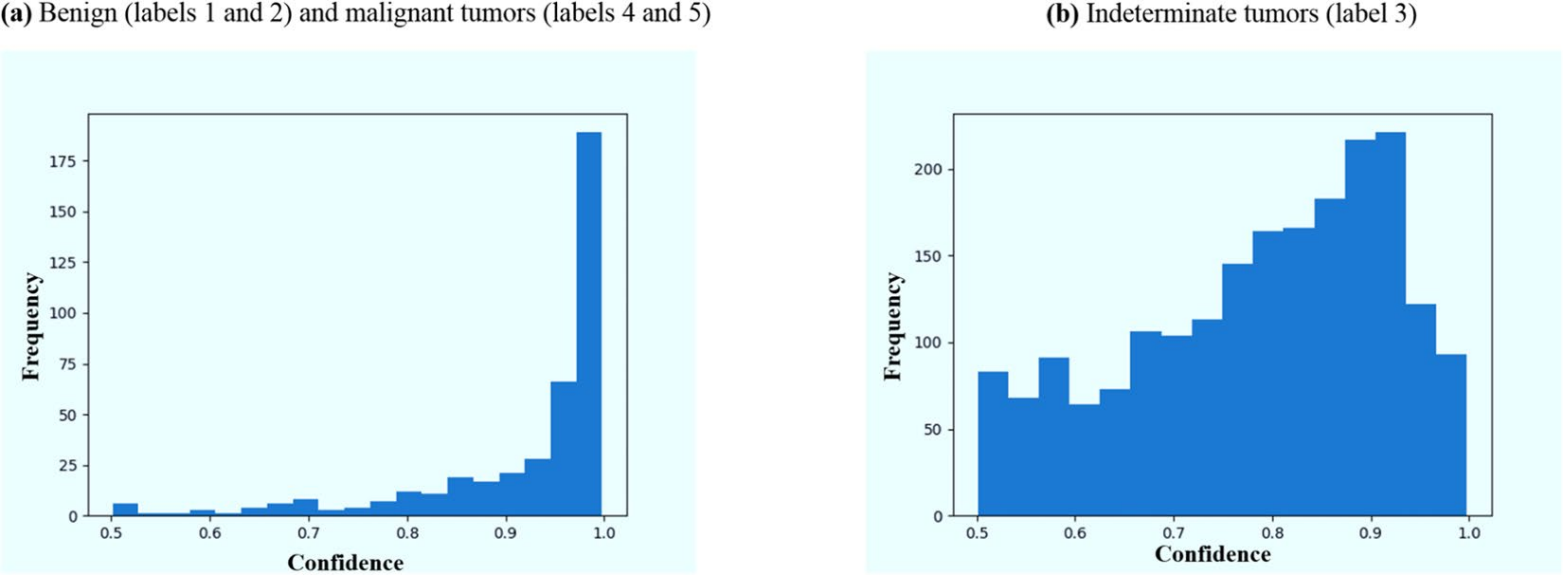
Histogram of the model’s confidence in classifying the tumors. 1 indicates a complete confidence, whereas 0.5 is a random assignment.

As stated previously, we discarded the tumors labeled as indeterminate (label 3). In practice, however, the model may still encounter these indeterminate cases. We investigated the output of the model when fed with these nodules. As shown in Fig. 6, while the model was relatively confident about benign and malignant nodules, it was mostly unconfident about the indeterminate ones, where confidence was defined as the probability the model assigns to the output class. This lack of confidence can be used as a sign that these cases should be referred to an expert for further examination.

There are two general paths to use the LIDC-IDRI dataset. The first one is to rely on the labels from the diagnosis data, which were obtained from different examinations including image review, biopsy, and surgical resection at a nodule level. This approach was explored by Kumar *et al*.^21^ for lung nodule malignancy prediction by training a CNN on the pathologically-proven diagnostic data and resulting in an accuracy of 77.52%, sensitivity of 79.06%, and specificity of 76.11%. A multi-view CNN for lung nodule classification, described by Liu *et al*.^39^, is another example of using the diagnostic data, that has achieved an AUC of 0.981 and error rate of 5.41%.

The second approach (also followed in our work) is to adopt the ratings provided by experienced radiologists at the time of reviewing the CT scans. Causey *et al*.^5^ applied a CNN based architecture to distinguish between benign and malignant nodules, and reported an AUC of 0.938, accuracy of 87.9%, sensitivity of 87.9%, and specificity of 87.9%. The authors have further improved the performance to an accuracy of 93.2% by incorporating the hand-crafted features. A Random Forest (RF) classifier was trained on the combination of hand-crafted and deep learning-based features to predict the nodule malignancy. Although the obtained accuracy was on a par with that of our proposed framework, it requires the nodules’ fine annotations, from which our model was completely independent. Table 3 presents a list of studies that have used the same setting of the LIDC-IDRI as we did, along with their proposed method and obtained results.

**Table 3:** A list of studies that have used LIDC-IDRI to predict lung nodule malignancy based on the ratings provided by radiologists. Note that some of the studies (refs.^5,32^) included hand-crafted features, requiring expert annotations.

Method	Area Under the Curve (AUC)	Accuracy	Specificity	Sensitivity
Proposed model	0.964	93.12%	90%	**94.94%**
CNN^5^	0.938	87.9%	87.9%	87.9%
CNN in combination with hand-crafted features^5^	**0.971**	**93.2%**	**98.5%**	87.9%
Deep residual network^30^	0.9459	89.90%	88.64%	91.07%
Deep belief network^31^	—	81.19%	—	—
CNN in combination with hand-crafted features^32^	—	86.79%	95.42%	60.26%
Multi-crop CNN^33^	0.93	87.14%	93%	77%

A significant challenge in comparing different studies on the LIDC-IDRI dataset is that different researchers have used different cohorts of training and testing. One solution to this challenge is to cross-validate the results, instead of using the fixed sets. This strategy, however, should be used with care, not to include nodules from the same patient in both sets. Furthermore, in the case of using deep learning models, such as the one we proposed in this study, cross validation can be computationally expensive. Another challenge of comparing the studies is the difference between reported performance measurements. Accuracy, which is the only metric provided in several studies, is not informative enough, as it gives no details on the portions of positive and negative samples, and a highly biased model can lead to a high accuracy. Our proposed 3D Multi-scale CapsNet model achieves high accuracy, as well as high sensitivity and specificity, showing that it is not biased towards positive or negative samples. Another limitation of most of the previous models is that they require a larger number of samples and they rely on heavy data augmentation. However, the model proposed here was based on the CapsNet, which was capable of interpreting small datasets. This study shows that good performance may be achievable even without large datasets, which is often the case in medical imaging.

In conclusion, the proposed 3D-MCN model, which was a multi-scale version of the capsule network, was shown to be advantageous over single-scale models, 3D-CNN, and previous studies on the LIDC-IDRI dataset. Although there is still a long way to go until deep learning-based radiomics can be reliably used in clinical applications, we believe the proposed model in this study was a big step forward to this end, by offering complementary information to guide lung nodule malignancy prediction.

## Methods

### Dataset description

The LIDC-IDRI is a collection of 1018 CT scans from 1010 patients. The nodules in this collection are identified and annotated through a two-phase process. In the first phase, 12 radiologists independently reviewed the scans and marked the lesions as nodule ≥3 mm, nodule ≥3 mm, and non-nodule. Radiologists annotated the ones identified as nodule ≥3 mm. In the second step, radiologists had access to the results of other radiologists to refine their own marks or leave them unchanged. After this phase, radiologists independently assessed several characteristics of the nodules ≥3 mm, including the likelihood of malignancy, shape, margin, and internal structure. Malignancy was rated from 1 to 5, where 1 indicates the lowest malignancy likelihood and 5 denotes the highest.

### Nodule patch selection and processing

In this work, we chose nodules ≥3 mm to classify them as benign (rating of 1 and 2) or malignant (rating of 4 and 5) based on ratings provided by radiologists in the LIDC-IDRI study. We included all the marked nodules, in either the training or the test set, even if the nodule was identified by only one radiologist, to have a model that is more robust to noisy inputs. Keeping these noisy samples was useful in the sense that it represented the overlap between the two distributions of nodule and non-nodule, which contributes to random error. The labels of the nodules that were identified by more than one radiologist were the average over all the available ratings, rounded to the nearest integer. Consequently, nodules with an average malignancy of 3 (indeterminate malignancy) were discarded.

For each nodule, we extracted three different 3D patches around the nodule center, where 3D patch refers to extracting one patch from the central slice, and two from the two immediate neighbors. Each 3D patch was extracted at three different scales. The first scale completely fits the nodule boundary, based on the provided annotation. As nodules are associated with different sizes, all extracted patches were zero padded up to the fixed size of 80 × 80 (the largest possible width and height based on the training data). The second scale was extracted by allowing a margin of 10 pixels at each side. The patches were zero padded to the fixed size of 100 × 100, and down-sampled, using the bi-linear method, to 80 × 80, to be consistent with the first scale and reduce the complexity. For down-sampling, we used the “bi-linear” approach, which is an extension of linear interpolation, where linear down-sampling (weighted average of the two immediate neighbors) was first performed in one direction, followed by the down-sampling in the other direction. Similarly, the third scale was extracted by allowing a margin of 20 pixels at each side. The patches were zero padded to the fixed size of 120 × 120 and down-sampled to 80 × 80. At the end, data was normalized between 0 and 1. The training set was shuffled and augmented by including random flipping. Finally, we ended up with three sets of training (at three scales), three sets of test, one set of training label, and one set of test label. Each training set was fed to an independent CapsNet along with its corresponding test set. It is worth mentioning that although increasing the number of scales would possibly improve the performance by capturing more detailed inter and intra-tumoral heterogeneity, it comes with extra computational overhead requiring advanced memory resources. Taking patches at three scales, therefore, is the commonly used approach^40^. While small patches offer more consistency, they cannot capture the object-whole relations. Large patches, on the other hand, lose the object details^41^. The three scales used in our study represent information from fine, medium, and coarse nodule boundaries^42^.

### Capsule network

CNNs have been the state-of-the-art in object recognition for many years. These networks, generally, benefit from the shared weights across the input, enabling them to detect local features with far less trainable parameters. However, the embedded pooling layers are shown to lose the exact information on spatial relations. Therefore, CNNs are not robust to transformation and rotation, and they need to be fed with all the possible inputs in order to learn the translations. CapsNets, on the other hand, are more capable of handling transformations and rotations through the capsule layers and “routing by agreement” process.

**Figure 7:**
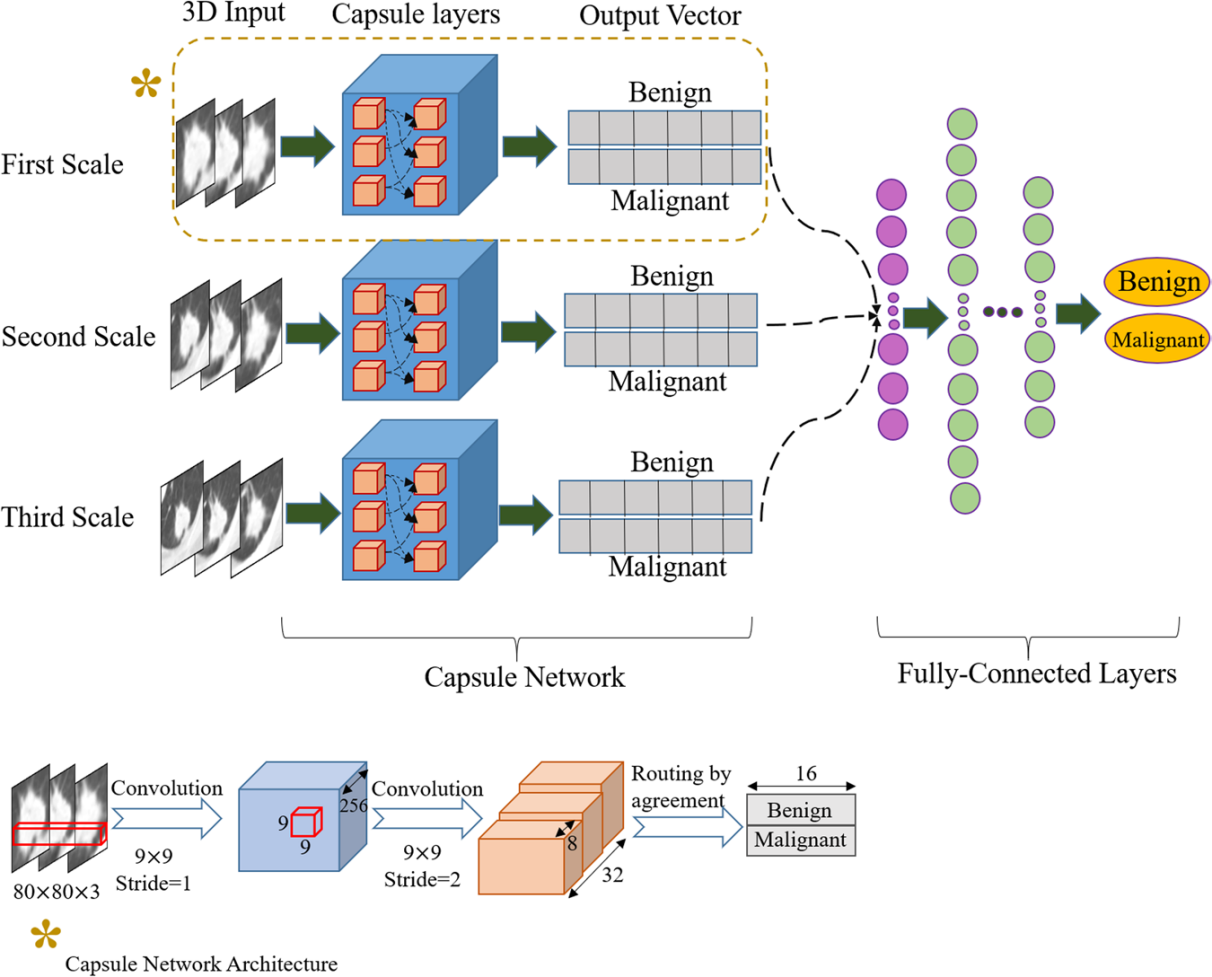
The proposed 3D multi-scale CapsNet framework. Three independent capsule networks take 3D nodule crops as inputs. Each CapsNet takes inputs at a different scale. The output vectors are masked and concatenated into a single vector. The resulted vector goes through a fusion module consisting of a set of fully connected layers to form probability associated with each class (benign or malignant). The CapsNet detailed architecture is marked with an asterisk at the bottom.

A CapsNet is a neural network consisting of several capsules. A capsule is a group of neurons that, together, predict the probability of presence and instantiation parameters of a specific object at a specific location. In contrast to CNNs, CapsNets preserve the spatial information throughout the network. The core process of a CapsNet is similar to that of conventional neural networks, where each neuron contributes to the output of the neurons in the layer above. Each capsule in a CapsNet predicts the output of all the capsules in the next layer. These predictions, however, are not treated equally, as in a conventional network. The contribution of each lower level capsule depends on its success in predicting the output of the next layer’s capsules. The process, through which the weights for the predictions are determined, is called “routing by agreement” and forms the main idea behind the CapsNets. This process enables the network to consider the spatial relations between the low level and high level instances, without looking at all possible translations and being fed a huge dataset. Since, large datasets are not typically accessible in medical fields, CapsNet is an appropriate choice for the problem of lung nodule classification. In general, a Capsule *i* predicts the output of the next layer’s Capsule *j*, denoted by ${\hat{{\bf{u}}}}_{j|i}$, where predictions are learned through a back-propagation process, the same as the learning approach in a conventional neural network, as follows

2 $${\hat{{\bf{u}}}}_{j|i}={{\bf{W}}}_{ij}{{\bf{u}}}_{i},$$

where **u**_*i*_ denotes the instantiation parameters of the capsule *i*, and **W**_*ij*_ is the weight matrix used for the prediction, learned through the back-propagation. This prediction process is similar to what happens in a CNN. The output of Capsule *j*, denoted by **s**_*j*_, is a weighted summation over all the incoming predictions (${\hat{{\bf{u}}}}_{j|i}$), with different weighting coefficients, which are learned thorough the routing by agreement procedure. Routing is what distinguishes the CapsNet from a CNN, and helps with identifying the spatial relations. This procedure can be summarized as follows

3 $${a}_{ij}={{\bf{s}}}_{j}.{\hat{{\bf{u}}}}_{j|i},\,\,{b}_{ij}={b}_{ij}+{a}_{ij},\,\,{c}_{ij}=\frac{exp({b}_{ij})}{\sum _{k}exp({b}_{ik})},\,\,{\rm{a}}{\rm{n}}{\rm{d}}\,{{\bf{s}}}_{j}=\sum _{i}{c}_{ij}{\hat{{\bf{u}}}}_{j|i},$$

where *a*_*ij*_ measures the degree of similarity between **s**_j_ and its prediction ${\hat{{\bf{u}}}}_{j|i}$, by calculating their inner product, and ***c***_*ij*_ denotes the coupling coefficient associated with the prediction. The final output ***s***_*j*_ typically goes through a non-linear squashing function to prevent it from exceeding one. Finally, it should be noted that the loss function specifically proposed for the CapsNet is slightly different from the common loss functions such as the cross-entropy. The CapsNet loss function, *l*_*k*_, associated with capsule *k*, is calculated as follows

4 $${l}_{k}={T}_{k}max{(\mathrm{0,}{m}^{+}-||{{\bf{s}}}_{k}||)}^{2}+\lambda (1-{T}_{k})max{(\mathrm{0,}||{{\bf{s}}}_{k}||-{m}^{-})}^{2},$$

where *T*_*k*_ is one whenever the class *k* is present and zero otherwise. Terms *m*^+^, *m*^−^, and *λ* are the hyper parameters of the model. Term **s**_*k*_ for each last layer Capsule *k* is associated with a particular class (benign or malignant), determining both the probability of the class and the instantiation parameters. The CapsNet-based model (3D-MCN) that we developed for malignancy prediction of lung nodules is shown in Fig. 7. In this framework, we have trained three independent CapsNets on three different scales, and obtained the **s**_*k*_ s for all three CapsNets and both output classes, on both the training and test set, to be fed to our multi-scale network. The CapsNet architecture that we used for a single scale is shown in Fig. 7 with an asterisk. This architecture consists of a convolutional layer, a primary capsule layer that predicts the output of the next layer, and a classification capsule layer that outputs the probability of each class along with the instantiation parameters (**s**_*k*_ s).

### Multi-scale model

Our multi-scale model was a fully connected neural network with 3 three hidden layers of sizes 1028, 512, and 256. The input to this network was the combination of all the output instantiation vectors from the three CapsNets. For each CapsNet, the output vector of the lower probability class was masked (set to zero). The CapsNet architecture is shown in Fig. 7 with an asterisk. Each output class is of dimension 16. Having two output classes (benign and malignant) results in a vector of dimension 32 for each CapsNet, and having three CapsNets results in an input of size 96 to the multi-scale network. The output of this network was the probability of the nodule being benign or malignant, based on the information from all the three scales.

We have implemented our model using the *Keras*^43^ software package. The multi-scale network objective was a cross entropy loss function optimized using the Adam optimizer.
